# Effects of Aerobic Exercise on Irisin and Skeletal Muscle Autophagy in *ApoE^−/−^* Mice

**DOI:** 10.3390/cimb47050371

**Published:** 2025-05-19

**Authors:** Wenxin Wang, Fengting Zheng, Jiawei Zhou, Yangfan Cao, Liang Zhang, Yao Lu, Qingbo Li, Ting Li, Mallikarjuna Korivi, Lifeng Wang, Wei Li

**Affiliations:** College of Physical Education and Health Sciences, Zhejiang Normal University, Jinhua 321004, China; 15068671301@163.com (W.W.); 15157060709@163.com (F.Z.); zhoujw110@zjnu.edu.cn (J.Z.); 2022207000835@zjnu.edu.cn (Y.C.); zhangliang0433@163.com (L.Z.); gbiyyl@163.com (Y.L.); lqb1971701428@163.com (Q.L.); tingli@zjnu.edu.cn (T.L.); mallik.k5@gmail.com (M.K.)

**Keywords:** atherosclerosis, skeletal muscle, aerobic training, autophagy, irisin

## Abstract

As a chronic inflammatory disease, atherosclerosis can affect the occurrence of skeletal muscle autophagy through a variety of mechanisms. Previous studies have demonstrated that exercise enhances autophagic activity through irisin-mediated pathways. Building upon this evidence, this study investigated the effects of a 12-week aerobic exercise training on irisin levels and skeletal muscle autophagy-related proteins in atherosclerotic mice. Male C57BL/6J and *ApoE^−/−^* mice were randomly assigned to four groups: Control Group (C), Aerobic Exercise Group (CE), *ApoE^−/−^* Control Group (AC), and *ApoE^−/−^* Aerobic Exercise Group (AE). Serum and muscle irisin levels were measured by ELISA; the expression levels of FNDC5, AMPK/mTOR pathway proteins and autophagy markers were detected by immunoblots, and muscle morphology was examined using H&E staining. Compared with the C group, the serum levels of TAG, TC, and LDL-C were higher than the AC group. Aerobic exercise increased irisin levels in skeletal muscle, upregulated the expression of LKB1 and p-AMPK, and presented an elevated LC3-II/I ratio, accompanied by reduced mTORC1 expression in CE mice. Aerobic exercise increased FNDC5 expression and irisin levels in serum and skeletal muscle, but also upregulated mTORC1 expression and reduced the LC3-II/I ratio in the AE group. Aerobic exercise enhances irisin synthesis and improves dyslipidemia in *ApoE^−/−^* mice. However, the increased expression of the mTORC1 protein contributed to decreasing the expression of autophagy-related proteins following exercise.

## 1. Introduction

Atherosclerosis (AS) is a chronic cardiovascular disease characterized by coronary and peripheral arterial dysfunction, leading to skeletal muscle ischemia, metabolic dysregulation and accompanied by impaired skeletal muscle performance [1,2]. Pro-inflammatory cytokines secreted by atherosclerotic plaques can trigger inflammatory signaling pathways and suppress the expression of autophagy-associated proteins, potentially contributing to skeletal muscle dysfunction [3].

Irisin, a myokine identified in 2012, is produced via the cleavage of the fibronectin type III domain—containing protein 5 (FNDC5) receptor [4]. Structurally, irisin comprises an N-terminal fibronectin III (FNIII)-like domain linked to a flexible C-terminal tail region. Notably, the FNIII-like domain adopts a continuous intersubunit β-sheet dimer conformation [5]. Emerging evidence suggests that irisin exerts protective effects against cardiovascular diseases, including AS, myocardial ischemia/reperfusion injury, and ventricular remodeling, by modulating inflammatory pathways and enhancing energy metabolism [6,7,8]. Aerobic exercise is a well-established intervention for AS, effectively reducing arterial plaque inflammation through improved lipid metabolism, suppression of inflammatory responses, and vascular function restoration, ultimately lowering cardiovascular disease incidence and mortality [9,10,11]. Exercise-induced increases in irisin levels may activate LKB1 or indirectly trigger AMPK phosphorylation via calcium-dependent signaling (e.g., the CaMKKβ pathway), thereby regulating skeletal muscle autophagy [12,13].

Autophagy is a fundamental physiological defense mechanism that employs lysosomal degradation to counteract pathological stimuli, maintaining cellular structure, function, and homeostasis [14]. Given the high metabolic demands of skeletal muscle, its autophagic activity is crucial for sustaining homeostasis and promoting cellular regeneration [15]. Studies suggest that AS may influence other tissues or organs by modulating autophagy-related pathways [16]. During exercise, increased AMP levels and decreased ATP levels activate AMPK, which suppresses mTORC1 activity and stimulates ULK1. Additionally, AMPK phosphorylates multiple downstream autophagy-related proteins, facilitating autophagosome formation and initiating the autophagic process [17].

We hypothesize that aerobic exercise may exert significant effects on skeletal muscle autophagy in ApoE^−/−^ mice through modulation of irisin levels, which may play a pivotal role in alleviating skeletal muscle disorders associated with atherosclerosis. Therefore, this study primarily analyzed irisin levels, AMPK/mTOR pathway proteins, and autophagy-related protein expressions after aerobic exercise.

## 2. Materials and Methods

### 2.1. Animal Care, Grouping, and Treatment

Twelve male C57BL/6J mice (6 weeks old) were randomly assigned to a control group (C, n = 6) or an aerobic exercise group (CE, n = 6). Similarly, 12 age-matched male apolipoprotein E knockout (*ApoE^−/−^*) mice were allocated to an atherosclerosis model control group (AC, n = 6) or an exercise intervention group (AE, n = 6). All animals were housed in a specific pathogen-free (SPF) facility at the Animal Experiment Center of Zhejiang Normal University under controlled conditions (temperature: 22 ± 2 °C; relative humidity: 55 ± 10%; 12-h light–dark cycle, lights on from 08:00 to 20:00).

To induce atherosclerosis, AC and AE mice were fed an HFD (60% kcal from fat, 20% from protein, 20% from carbohydrates), while C and CE mice received standard chow (10% kcal from fat, 20% from protein, 70% from carbohydrates). All experimental procedures were reviewed and approved by the Institutional Animal Care and Use Committee of Zhejiang Normal University (Protocol Approval No. ZSDW2024025), in strict accordance with the relevant regulations of the National Laboratory Animal Welfare Ethics Committee.

### 2.2. Exercise Protocol

Mice in the CE and AE groups underwent 12 weeks of treadmill training. Before starting the full exercise protocol, mice in the exercise groups underwent a 1-week adaptation to rodent treadmill training. On Day 1, they were introduced to the treadmill at 10 m/min for 10 min. From Day 2, speed and duration rose by 1 m/min and 10 min daily until hitting 15 m/min for 60 min. The final protocol involved running at 15 m/min for 60 min with a 5° incline, allowing 2 min rests every 15 min. Training took place from 5 pm to 7 pm, Monday to Friday, for 12 weeks, at an intensity of approximately 60% of the mice’s maximal oxygen uptake [18,19].

### 2.3. Skeletal Muscle Tissue Sampling

After a 24-h fast, mice were anaesthetized on the third day and subsequently dissected. Skeletal muscle samples, including the quadriceps femoris muscle, tibialis anterior muscle, and extensor digitorum longus muscle, were excised, rinsed in cold phosphate-buffered saline (PBS), blotted dry, and weighed to record wet weight. The samples were then labelled, snap-frozen in liquid nitrogen, and stored at −80 °C. For the CE and AE groups, tissue collection occurred 48 h after the final exercise session. Animal carcasses were temporarily stored at −80 °C before transfer to the animal facility for disposal via certified incineration.

### 2.4. Measurement of Lipid Levels

Blood was collected from the abdominal aorta and centrifuged at 3000 rpm for 20 min at 4 °C to isolate serum, which was subsequently stored at −80 °C until analysis. Serum triacylglycerides (TAG), total cholesterol (TC), low-density lipoprotein cholesterol (LDL-C), and high-density lipoprotein cholesterol (HDL-C) levels were quantified using commercial assay kits (Nanjing Jiancheng Bioengineering Institute, Nanjing, China) following the manufacturer’s instructions. Absorbance was measured using a microplate reader (A51119700DPC, Thermo Fisher Scientific, Waltham, MA, USA) at 510 nm (TAG, TC) and 546 nm (LDL-C and HDL-C).

### 2.5. Detection of Irisin in Skeletal Muscle and Serum

Irisin levels in skeletal muscle and serum were quantified using a mouse irisin ELISA kit (RX202296M, Ruixin Biotechnology, Quanzhou, China). Following the manufacturer’s protocol, microplates pre-coated with specific capture antibodies bound the target protein in samples, which subsequently formed a sandwich complex with biotinylated detection antibodies and enzyme-conjugated streptavidin-HRP. After TMB chromogenic reaction, absorbance was measured at 450 nm using a microplate reader (Thermo Fisher Scientific, Waltham, MA, USA), and irisin concentrations in skeletal muscle and serum samples were quantified via standard curves. The experiment included sample pretreatment, incubation for antigen-antibody binding, washing steps, chromogenic development, and data calculation.

### 2.6. Hematoxylin-Eosin (HE) Staining

Quadriceps femoris muscle samples were rinsed in pre-cooled PBS, fixed in paraffin, and sectioned using a microtome. Tissue sections were deparaffinized with two changes of xylene (5 min each) and rehydrated through a graded ethanol series (100%, 95%, 90%, and 75%). Sections were stained with hematoxylin for 15 min, rinsed in running tap water until blue, and counterstained with eosin for 30 s. Dehydration and clarification were performed using sequential immersion in 95% ethanol (twice, 1 min each), anhydrous ethanol (twice, 10 s each), and xylene (twice, 1 min each). Coverslips were mounted with glycerol gelatine for microscopic examination. Cross-sectional areas were analyzed using ImageJ software (Ver 1.51-java 8).

### 2.7. Immunoblots

Proteins were extracted and quantified from the quadriceps femoris muscle, tibialis anterior muscle, and extensor digitorum longus muscle tissues. Equal amounts of protein (20 μg per lane) were separated via SDS-PAGE and transferred onto PVDF membranes using transfer buffer (14.63 g glycine, 3.03 g Tris, 200 mL methanol, 800 mL deionized water). Membranes were blocked with 5% non-fat milk in 1× TBST and incubated overnight at 4 °C with primary antibodies at the following dilutions: FNDC5(1:1000, 82671-1RR, Proteintech, Rosemont, IL, USA), LC3B (1:1000, #2775, Cell Signalling Technology, Danvers, MA, USA), p62 (1:5000, 18420-1-AP, Proteintech, Rosemont, IL, USA), AMPK (1:1000, #5832, Cell Signalling Technology, Danvers, MA, USA), p-AMPK (1:1000, #2535, Cell Signalling Technology, MA, USA), mTOR (7C10) (1:1000, #2983, Cell Signalling Technology, MA, USA), LKB1 (1:500, 10746-1-AP, Proteintech, Rosemont, IL, USA), and GAPDH (1:50000, 60004-1-Ig, Proteintech, Rosemont, IL, USA). Following washes with 1× TBST, membranes were incubated with HRP-conjugated secondary antibodies for 2 h at room temperature, washed again, and developed using an enhanced chemiluminescence system. Protein band intensities were quantified using Image Lab 6.1 software and normalized to GAPDH.

### 2.8. Statistical Analysis

All experimental data were analyzed using ImageJ software (Ver 1.51-java 8, Bethesda, MD, USA), and graphs were generated with GraphPad Prism 9.5 (Version 9.5, San Diego, CA, USA). Statistical analyses were conducted using one-way ANOVA, with homogeneity of variance assessed prior to testing. Post hoc comparisons were performed using Tukey’s test. Results are presented as mean ± SD, with statistical significance set at α = 0.05 (*: *p* < 0.05; **: *p* < 0.01; ***: *p* < 0.001).

## 3. Results

### 3.1. Exercise Attenuates Dyslipidemia in ApoE^−/−^ Mice

**Figure 1:**
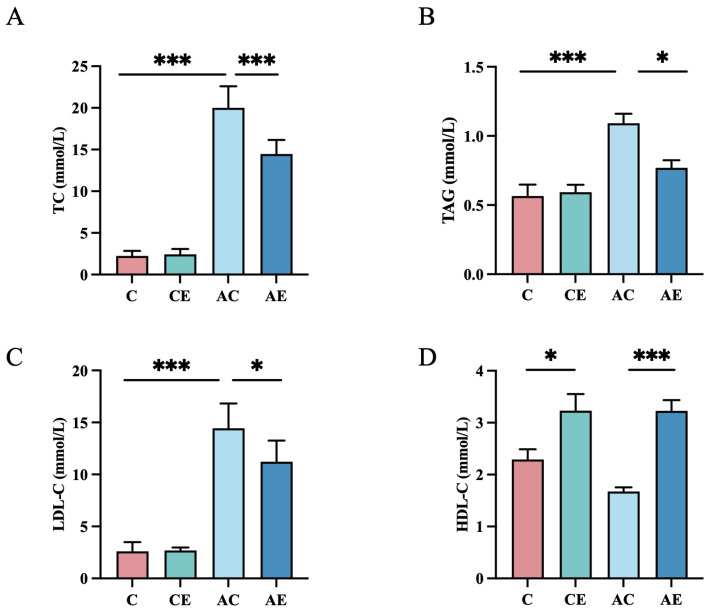
Aerobic exercise decreased plasma lipid levels in *ApoE^−/−^* mice. (**A**–**D**) Plasma TC, TAG, LDL-C, and HDL-C levels of mice. Data presented as mean ± SD. * *p* < 0.05; *** *p* < 0.001.

As shown in Figure 1, the AC group exhibited significantly higher TAG, TC, and LDL-C levels compared to the C group (*p* < 0.001). In AE mice, aerobic exercise significantly reduced TC (*p* < 0.001), TAG (*p* < 0.05), and LDL-C (*p* < 0.05), while increasing HDL-C (*p* < 0.01) compared to the AC group. These findings indicate that 12 weeks of aerobic exercise significantly improves dyslipidemia in atherosclerotic mice.

### 3.2. Exercise Exerted No Significant Influence on the Cross-Sectional Fiber Area Fibers in ApoE^−/−^ Mice

**Figure 2:**
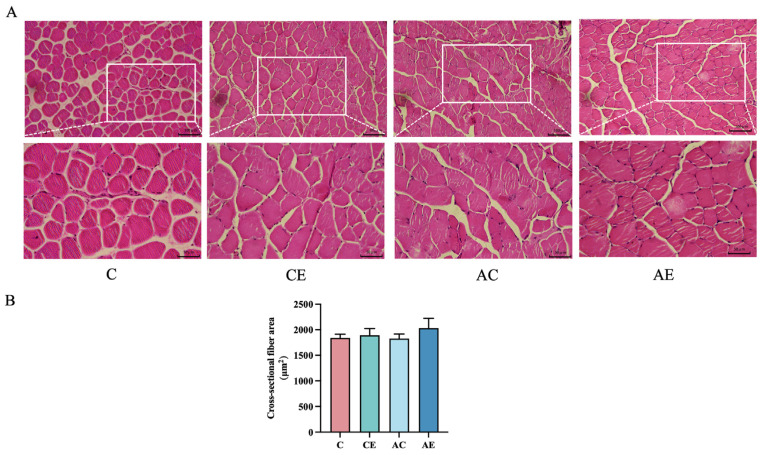
Aerobic exercise on cross-sectional fiber area in wild mice and *ApoE^−/−^* mice. (**A**,**B**) Slides of skeletal muscle tissue were prepared with HE staining. Digital images were captured using 20× and 40× magnification (upper panel). Scale bar = 100/50 µm. Data presented as mean ± SD.

HE staining was performed to assess the effect of aerobic exercise on skeletal muscle fiber morphology in *ApoE^−/−^* mice. As shown in Figure 2, 12 weeks of aerobic exercise did not significantly alter the cross-sectional fiber area in either WT or *ApoE^−/−^* mice.

### 3.3. Exercise Increased Irisin Levels in ApoE^−/−^ Mice

**Figure 3:**
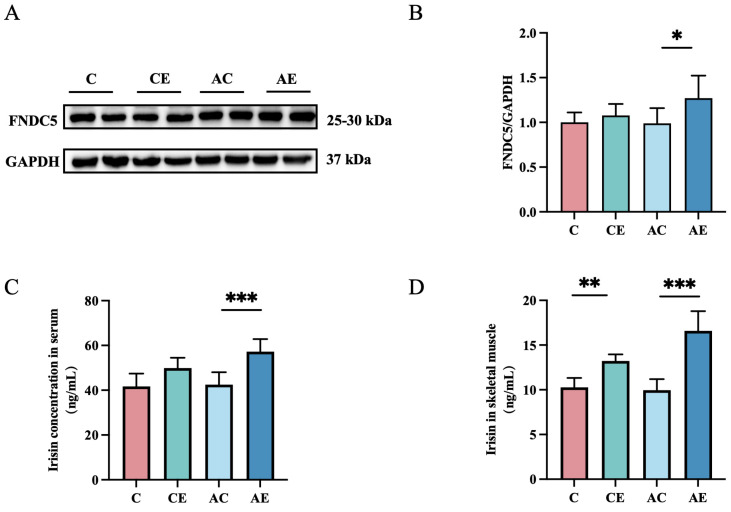
Aerobic exercise promotes irisin production in *ApoE^−/−^* mice. (**A**,**B**) Immunoblots for FNDC5 protein expression in the quadriceps femoris muscle of mice. (**C**) Irisin concentrations in the serum of mice. (**D**) Irisin concentrations in the skeletal muscle of mice. Data presented as mean ± SD (n = 6). * *p* < 0.05; ** *p* < 0.01; *** *p* < 0.001.

The effect of aerobic exercise on irisin levels remains controversial. As shown in Figure 3, exercise significantly increased skeletal muscle irisin levels in CE mice (*p* < 0.05). Compared to the AC group, the expression of irisin in both serum (*p* < 0.01) and skeletal muscle (*p* < 0.001) was significantly upregulated in the AE group. Moreover, the expression of FNDC5 (*p* < 0.05), a precursor protein of irisin, was also significantly increased. These results suggest that 12 weeks of aerobic exercise promotes irisin production in both serum and skeletal muscle of *ApoE^−/−^* mice.

### 3.4. Exercise Upregulated Protein Expression of mTORC1, but Had No Effect on the APMK in ApoE^−/−^ Mice

**Figure 4:**
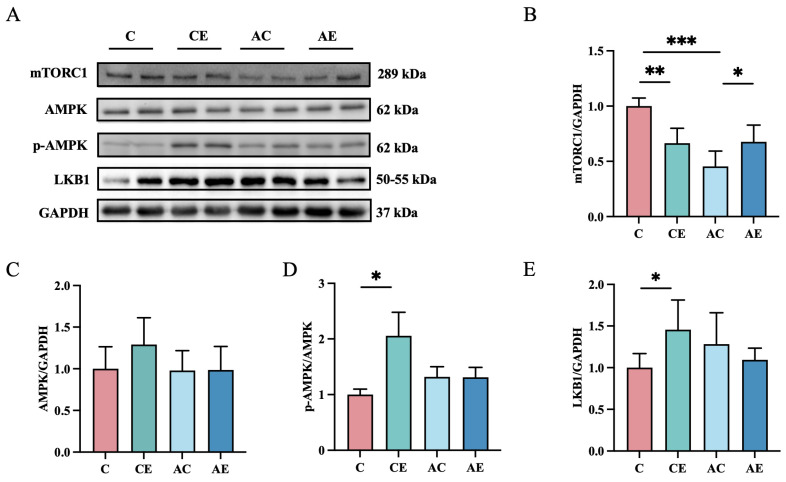
Aerobic exercise did not have a particularly large effect on the AMPK/MTOR pathway in *ApoE^−/−^* mice. (**A**–**E**) Immunoblots for mTORC1, AMPK, p-AMPK, and LKB1 protein expression in the quadriceps femoris muscle of mice. Data presented as mean ± SD (n = 6). * *p* < 0.05; ** *p* < 0.01; *** *p* < 0.001.

To investigate the effects of irisin on the AMPK/mTOR pathway, protein levels were analyzed (Figure 4). In CE mice, exercise upregulated LKB1 (*p* < 0.05) and p-AMPK (*p* < 0.05), while downregulated mTORC1 expression (*p* < 0.01). However, in AE mice, no significant changes were observed in AMPK or p-AMPK expression, whereas mTORC1 expression was significantly increased (*p* < 0.05).

### 3.5. Exercise Inhibits the Expression of Autophagy-Related Proteins in Skeletal Muscle of ApoE^−/−^ Mice

**Figure 5:**
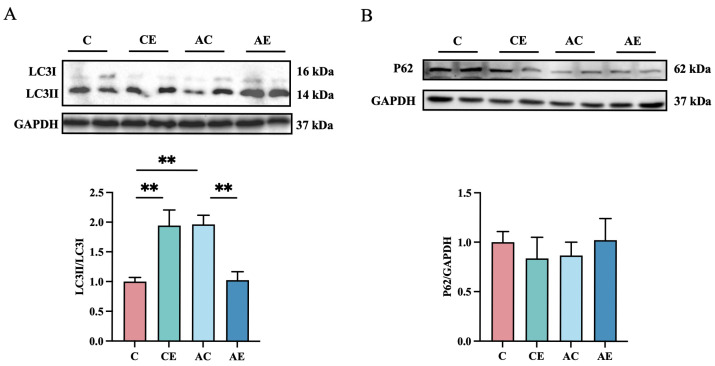
Aerobic exercise decreased autophagy-related proteins in *ApoE^−/−^* mice. (**A**,**B**) Immunoblots for LC3 and P62 protein expression in quadriceps femoris muscle of mice. Data presented as mean ± SD (n = 6). ** *p*< 0.01.

To assess the impact of exercise on autophagy, the expression of p62 and LC3 I/II proteins was examined (Figure 5). No significant differences in p62 expression were observed among the groups. Compared to the C group, the LC3 II/LC3 I ratio was significantly increased in the AC group (*p* < 0.01). Aerobic exercise further enhanced the LC3 II/LC3 I ratio in the CE group (*p* < 0.01), whereas a significant reduction (*p* < 0.01) in this ratio was observed in the AE group following exercise.

## 4. Discussion

Our findings demonstrated that 12 weeks of aerobic exercise training significantly improved lipid abnormalities, as evidenced by reduced serum TAG, TC, and LDL-C levels, along with increased HDL-C in *ApoE^−/−^* mice. Exercise training restored the irisin concentrations in both serum and skeletal muscle of *ApoE^−/−^* mice. However, the reduced LC3-II/I ratio, accompanied by reduced autophagy-related protein expressions, contradicted our initial hypothesis, a finding that might be associated with the upregulation of mTORC1. These findings support the potential of aerobic exercise as a nonpharmacologic intervention for combating dyslipidemia.

Atherosclerosis induces significant alterations in lipid metabolism and traditional lipid profile, and a dyslipidemic state reciprocally accelerates atherosclerotic progression [20]. In atherosclerotic patients, impaired LDL receptor functionality has been associated with prolonged circulatory retention of LDL-C, enhanced susceptibility to oxidative modification, and subsequent macrophage-mediated internalization, which collectively drive foam cell formation and plaque advancement [21]. Irisin has been shown to attenuate plasma LDL-C and TAG levels, improve lipid homeostasis, and suppress lipid peroxidation, thereby mitigating atherosclerotic plaque formation in *ApoE^−/−^* mice [22]. The experimental findings of this study are consistent with findings by Li et al. [23], indicating that irisin may serve as a potential therapeutic agent for mitigating atherosclerosis and its associated skeletal muscle damage. Further research is warranted to explore the underlying mechanisms and clinical applications of irisin in this context.

Interestingly, our results indicate that 12 weeks of aerobic exercise did not significantly alter the cross-sectional fiber area in mice. This finding contrasts with previous studies reporting aerobic exercise-induced adaptations in skeletal muscle, including increased myofiber cross-sectional area (CSA) [24,25]. The discrepancy may be attributed to murine-specific adaptive responses, potentially influenced by differences in exercise modality, intensity, or species-specific physiological mechanisms. It is possible that the exercise regimen employed in this study remained within the physiological tolerance thresholds of the mice, failing to provide sufficient mechanical overload to induce significant morphological alterations in skeletal muscle architecture. From an energy metabolism perspective, the mice may have maintained energy homeostasis during the 12-week aerobic exercise regimen through alternative metabolic pathways rather than CSA expansion. As demonstrated by Ouyang et al. [26], energy-demanding conditions, such as exercise, promote triglyceride hydrolysis within lipid droplets, releasing free fatty acids for mitochondrial β-oxidation to sustain cellular energetics. This metabolic adaptation suggests a preferential reliance on lipid mobilization to meet the energy demands of exercise, thereby reducing the necessity for skeletal muscle hypertrophy. Such substrate utilization plasticity may explain the absence of significant CSA modifications despite prolonged aerobic training.

Irisin, an exercise-induced myokine, promotes its synthesis by stimulating FNDC5 expression in response to exercise or other metabolic stresses. It subsequently activates AMPK-related downstream signaling targets to regulate autophagy [27,28]. Guo [29] demonstrated that irisin-knockout (irisin-KO) mice exhibited exacerbated age-induced muscle atrophy and progressive sarcopenic features, while chronic intraperitoneal administration of recombinant irisin effectively attenuated age-related muscle atrophy and metabolic disorders in aged mice, highlighting the critical role of irisin in maintaining muscle physiology and systemic energy homeostasis during aging. Our experimental results demonstrate that a 12-week aerobic exercise intervention significantly upregulated irisin synthesis in the skeletal muscle of *ApoE^−/−^* mice. These findings align with established phenotypic profiles observed in WT murine models and are supported by extensive preclinical research [30], further confirming that exercise enhances irisin production. Additionally, 12 weeks of aerobic exercise significantly activated p-AMPK in the CE group, accompanied by a significant increase in the LC3-II/LC3-I ratio, consistent with the findings of Botella et al. [31]. This suggests that aerobic exercise promotes autophagy through irisin-mediated AMPK activation.

However, despite the increase in skeletal muscle irisin levels in *ApoE^−/−^* mice, p-AMPK expression remained unchanged. Simultaneously, mTORC1 expression increased, and the LC3-II/LC3-I ratio significantly decreased, indicating suppressed autophagic activity. Mechanistically, mTOR, a key negative regulator of autophagy [15], exhibits complex exercise-dependent modulation. Under certain physiological conditions, exercise suppresses mTOR activity via AMPK-mediated energy-sensing pathways triggered by increased metabolic demand. However, in *ApoE^−/−^* mice, exercise-induced mTOR activation has been observed, likely due to their distinct metabolic profiles and dysregulated signaling cascades. For instance, exercise may activate mTORC1 through the PI3K/Akt signaling pathway [32], which inhibits autophagy by suppressing the kinase activity of the ULK1 complex [33]. Activated mTORC1 further impairs autophagosome formation and may downregulate AMPK activity, ultimately reducing autophagy. These findings underscore the potential regulatory role of the irisin-AMPK-autophagy signaling axis, though further investigation is required to elucidate the precise molecular mechanisms involved.

We hypothesize that tissue-specific autophagic dysregulation in *ApoE^−/−^* models may underlie the divergent baseline autophagic profiles observed in skeletal muscle. And the pathological characteristics of *ApoE^−/−^* mice may similarly lead to dysregulated autophagy in skeletal muscle, manifesting as excessive autophagic activity. However, aerobic exercise intervention may activate compensatory regulatory mechanisms that attenuate excessive skeletal muscle autophagy, thereby preserving muscle function. These findings suggest that exercise-induced autophagy may serve as a protective compensatory mechanism to counteract pathological hyperactivation in *ApoE^−/−^* models. Nevertheless, the precise molecular pathways underlying this phenomenon remain unclear. Future studies should focus on investigating skeletal muscle autophagy in *ApoE^−/−^* mice to elucidate its role in disease progression and therapeutic potential.

## 5. Limitations of the Study

In this study, we employed immunoblotting to quantify autophagy-related proteins (LC3 and p62). However, only LC3 displayed significant alterations, whereas p62 levels remained unaltered. To address this limitation, future investigations should integrate complementary methodologies—including immunofluorescence, and transmission electron microscopy (TEM)—to provide a more comprehensive and precise evaluation of autophagy activity.

## 6. Conclusions

A 12-week aerobic exercise intervention significantly upregulates irisin synthesis and alleviates dyslipidemia in *ApoE^−/−^* mice. However, in these mice, impaired AMPK activity and compensatory irisin responses may contribute to suppressed autophagy-related protein expressions. These findings highlight the dual role of exercise in maintaining metabolic homeostasis and regulating autophagy-related protein expressions while emphasizing the need for further mechanistic exploration of mTORC1-driven autophagy inhibition in *ApoE^−/−^* models.

## Data Availability

The datasets supporting the findings of this study are accessible from the corresponding author upon formal request.

## Abbreviations

The following abbreviations are used in this manuscript, listed in descending order of prominence: (Primary Detection Indicators: FNDC5, AMPK, p-AMPK, mTORC1, LKB1, and LC3; Secondary Detection Indicators: TC, TAG, LDL-C, HDL-C)

Abbreviations	Full Term
FNDC5	Fibronectin type III domain containing protein 5
AMPK	Adenosine 5′-monophosphate (AMP)-activated protein kinase
p-AMPK	phosphorylated AMP-activated protein kinase
mTOR	mechanistic Target of Rapamycin
mTORC1	mammalian target of rapamycin complex 1
LKB1	Serine-Threonine Kinase 11
LC3	Low Complexity Communication Codec
LC3 I	Microtubule-associated protein 1 light chain 3B I
LC3 II	Microtubule-associated protein 1 light chain 3B II
P62	Sequestosome-1 (SQSTM1)
TC	Total Cholesterol
TAG	Triacylglycerides
LDL-C	Low-Density Lipoprotein Cholesterol
HDL-C	High-Density Lipoprotein Cholesterol
AMP	Adenosine Monophosphate
ATP	Adenosine Triphosphate
ULK1	Unc-51 like autophagy activating kinase 1
SPF	Specific Pathogen Free
PBS	Phosphate Buffered Saline
SDS-PAGE	Sodium Dodecyl Sulfate-Polyacrylamide Gel Electrophoresis
PVDF	Polyvinylidene Fluoride
TBST	Tris Buffered Saline with Tween-20
CSA	Cross-sectional area
